# Exhalative Breath Markers Do Not Offer for Diagnosis of Interstitial Lung Diseases: Data from the European IPF Registry (eurIPFreg) and Biobank

**DOI:** 10.3390/jcm8050643

**Published:** 2019-05-09

**Authors:** Ekaterina Krauss, Maike Froehler, Maria Degen, Poornima Mahavadi, Ruth C. Dartsch, Martina Korfei, Clemens Ruppert, Werner Seeger, Andreas Guenther

**Affiliations:** 1 European IPF Registry & Biobank (eurIPFreg/bank), 35394 Giessen, Germany; ekaterina.krauss@hotmail.com (E.K.); maike.froehler@yahoo.com (M.F.); Poornima.Mahavadi@innere.med.uni-giessen.de (P.M.); Martina.Korfei@neuro.med.uni-giessen.de (M.K.); clemens.ruppert@innere.med.uni-giessen.de (C.R.); (werner.seeger@innere.med.uni-giessen.de (W.S.); 2 Universities of Giessen and Marburg Lung Center (UGMLC), Member of the German Center for Lung Research (DZL), 35394 Giessen, Germany; ruth.dartsch@innere.med.uni-giessen.de; 3 Agaplesion Lung Clinic, 35753 Greifenstein, Germany; o.maurer@klinik-waldhof.de; 4 Cardio-Pulmonary Institute (CPI), EXC 2026, Project ID: 390649896, Justus-Liebig University Giessen, 35394 Giessen, Germany

**Keywords:** idiopathic pulmonary fibrosis (IPF), exhalative breath markers, European Registry for idiopathic pulmonary fibrosis (eurIPFreg), interstitial lung diseases (ILD)

## Abstract

*Background*: New biomarkers are urgently needed to facilitate diagnosis in Interstitial Lung Diseases (ILD), thus reducing the need for invasive procedures, and to enable tailoring and monitoring of medical treatment. *Methods*: In this study we investigated if patients with idiopathic pulmonary fibrosis (IPF; *n* = 21), non-IPF ILDs (*n* = 57) and other lung diseases (chronic obstructive pulmonary disease (COPD) *n* = 24, lung cancer (LC) *n* = 16) as well as healthy subjects (*n* = 20) show relevant differences in exhaled NO (FeNO; Niox MINO), or in eicosanoid (PGE2, 8-isoprostane; enzyme-linked immunosorbent assay (ELISA)) levels as measured in exhaled breath condensates (EBC) and bronchoalveolar lavage fluids (BALF). *Results*: There was no significant difference in FeNO values between IPF, non-IPF ILDs and healthy subjects, although some individual patients showed highly elevated FeNO. On the basis of the FeNO signal, it was neither possible to differentiate between the kind of disease nor to detect exacerbations. In addition, there was no correlation between FeNO values and lung function. The investigation of the eicosanoids in EBCs was challenging (PGE2) or unreliable (8-isoprostane), but worked out well in BALF. A significant increase of free 8-isoprostane was observed in BALF, but not in EBCs, of patients with IPF, hypersensitivity pneumonitis (HP) and sarcoidosis, possibly indicating severity of oxidative stress. *Conclusions*: FeNO-measurements are not of diagnostic benefit in different ILDs including IPF. The same holds true for PGE2 and 8-isoprostane in EBC by ELISA.

## 1. Introduction

Interstitial Lung Diseases (ILD) in general, and idiopathic pulmonary fibrosis (IPF) in particular, are complex disorders with different pathogenetic pathways, heterogenous disease behavior, with great variability in disease severity and rate of progression, as well as different responses to treatment [1]. IPF is a progressive ILD with still unresolved causes; with a median survival of 3–5 years following diagnosis, IPF represents the most aggressive ILD, which is characterized by a progressive decline in lung function and quality of life [2,3]. According to a currently favored concept, IPF is a “two-hit” disease, in which a genetic predisposition affecting epithelial homeostasis meets environmental stressors, including exposure to known noxious dusts and fumes (e.g., asbestos, silica, outdoor pollution, and cigarette smoke) [4,5].

Safe diagnosis of ILD’s can be notoriously difficult, since the number of differential diagnoses is usually large and the clinical and radiographic (high-resolution computed tomography—HRCT) phenotype patterns can be incomplete or overlapping [6,7]. For this reason invasive procedures such as cryobiopsy during bronchoscopy or open lung biopsy are regularly needed, and are crucial for setting the diagnosis, but are also prone to serious side effects including bleeding, pneumothorax, triggering of exacerbation and death [8].

International consensus guidelines recommend that the diagnosis of IPF should be made at a multi-disciplinary level, in order to increase the specificity of the diagnostic process, especially in those cases with overlapping or incomplete patterns [9]. Hence, there is a great need for novel and specific, non-invasive diagnostic methods. Reliable, sensitive, and objective diagnostic and prognostic biomarkers could allow distinction between different forms of ILD and assessment of the risk of deterioration [10]. Up to now, however, there is no such biomarker in clinical routine use (except HRCT) to support such diagnostic process.

Biomarker research has been undertaken using peripheral blood, bronchoalveolar lavage fluid (BALF), and exhaled breath condensate (EBC) [11]. Numerous molecules involved in alveolar epithelial cell injury, proliferation and matrix remodeling as well as immune regulation have been proposed as potential biomarkers [10]. As example, in previous studies in IPF, epithelial proteins such as KL-6, surfactant protein (SP)-A, SP-D and other factors, e.g., indicating oxidative stress (e.g., serum hydroperoxide), have been suggested as biomarkers able to discriminate between stable and progressive disease (e.g., CCL18 in serum) or to indicate response to therapy (e.g., Toll interacting protein) [12,13,14,15]. Likewise, tissue samples based biomarkers, such as the number of fibroblast foci, the level of Ki-67(a marker of tissue proliferation), and caspase-3 (a marker of tissue apoptosis) were found to have prognostic implications [16]. 

However, there is currently no established biomarker in routine use for diagnosis and assessment of prognosis in ILD, and this holds particularly true for non-invasive markers. With regard to non-invasive markers, one pathway of interest for non-invasive detection is the free radical nitric oxide (NO), which is regulated by three isoforms of nitric oxide synthase (NOS), neuronal NOS, inducible NOS and endothelial NOS (eNOS). Several reports showed that NO plays an important role in the development of pulmonary fibrosis [17,18]. NO is an important physiological and pathophysiological messenger that belongs to the reactive nitrogen species and acts in the pulmonary system as a vaso- and bronchodilatory neurotransmitter, also showing some inflammatory properties and being a marker of oxidative stress. The fractionated exhaled nitric oxide (FeNO) can be detected with commercially available portable devices [19].

FeNO has been shown to be useful for the diagnosis of eosinophilic airway inflammation especially in asthma, and for determining steroid responsiveness in chronic respiratory inflammation, with a focus on avoiding unnecessary steroid therapies [20,21,22]. With regard to ILDs, some investigations have already been carried out. For example, increased FeNO values had been previously demonstrated in hypersensitivity pneumonitis (HP) and IPF patients [23].

Other volatile substances of potential interest are prostaglandin E2 (PGE2) and 8-isoprostane. PGE2 is a known antifibrotic mediator that plays an important role in pathogenesis of wound resolution and also been shown to be an important negative regulator of fibroblast activation and collagen expression released by an intact alveolar epithelium [24,25]. Inhalation of PGE2 in combination with selected small interfering ribonucleic acid (siRNA(s)) was recently proposed in some experimental studies for the treatment of IPF [19]. Additionally, 8-isoprostane acts a marker for oxidative stress, in which case the substance is produced by peroxidation of arachidonic acid. This biomarker shows biological activity as a pulmonary and renal vasoconstrictor. It has also been shown to contract human smooth muscle cells from the lungs in vitro and to increase platelet activity [26,27]. Some studies have been done on 8-isoprostane in ILD patients, but its role as a marker of oxidative stress remains unknown [28].

In the present study we therefore evaluated the diagnostic potential of FeNO, PGE2 and 8-isoprostane in EBCs in different ILDs and other lung diseases. Unfortunately, none of these markers turned out to have any diagnostic or prognostic potential.

## 2. Experimental Section

### 2.1. Clinical and Lab Data Collection 

In this study, a total of 138 patients (of them 21 IPF, 57 other ILD, 24 chronic obstructive pulmonary disease (COPD), 16 lung cancer (LC) and 20 healthy controls (HC)) from the Giessen site of the European IPF registry (eurIPFreg), have been analyzed on the basis of clinical data as well as obtained biomaterials.

The eurIPFreg is an Internet-based, multicentre registry linked to the European IPF Biobank (eurIPFbank). The data protection concept was accepted by local and national networks such as the TMF (Technology, Methods, and Infrastructure for Networked Medical Research e.V.) and official authorities (e.g., Hessian Data Protection Officer, Protocol Nr. 412101 from 25.08.2008). Both, eurIPFreg and eurIPFbank were permitted by institutional review boards in Germany and Europe (e.g., Ethics Committee of Justus-Liebig-University of Giessen; 111/08). The research was conducted strictly according to the principles of the Declaration of Helsinki. The eurIPFreg and eurIPFbank are listed in ClinicalTrials.gov (NCT02951416).

Patients above 18 years with IPF, other ILD’s, or other non-ILD lung diseases (as disease controls), who signed the informed consent, were selected for this study. The diagnosis of IPF was based on the American Thoracic Society (ATS) criteria [9].

The clinical data were collected at the time of enrolment (baseline, which usually also would be at the time of their first presentation in our clinic) and in intervals of 3 to 12 months thereafter (follow up) via patient and physician baseline and follow up questionnaires, which in total comprised of 1700 variables [2]. This study presents a consecutive patient’s cohort, therefore their medication was not withheld prior to the measurements. This report only contains data from eurIPFreg patients recruited at the Giessen site.

Biological materials such as blood, bronchoalveolar lavage fluid (BALF), tissue samples as well as exhaled breath condensates and EBCs were centrally managed through generation of patient-, time-, and specimen-specific Lab IDs; they were stored in the centralized European IPF Biobank (eurIPFbank) located in Giessen, Germany. Patients were asked to withdraw from food and nicotine intake a few hours prior to the measurements, as well as to rinse the mouth before analyses. A nose clip was consistently used. The FeNO measurements were carried out with the device NIOX Mino (Aerocrine, Bad Homburg, Germany). EBCs were collected according to the recommendations of the ATS/European Respiratory Society (ERS) Working Group with the turbo-DECCS device (ItalChill, Parma, Italy) [29]. The turbo-DECCS device has a thermoelectric module that works through the Peltier effect. The exhaled breath of the patient was passed through disposable polyethylene tubes into the collection tube of the device and cooled there. The DECCS polyethylene tube system (ItalChill, Parma, Italy) was changed for each patient. After the collection, the EBC was quickly filled into cryocups and frozen at −80 °C.

The quantitative detection of PGE2 and 8-Isoprostan in biomaterials was performed using a commercially available immunosorbent assay (ELISA, Cayman Chemical Company, Ann Arbor, MI, USA). It is based on an enzymatic immune assay (EIA) principle. In case of BALF, samples were directly transferred to the wells of the plate. In case of EBCs, we analyzed EBC fluid directly, as well as after concentrating its content. For this purpose, 600 μL of each sample were lyophilized in the vacuum centrifuge and dissolved thereafter in distilled water at a volume of 120 µL.

### 2.2. Quality of Data and Statistical Analysis

Quality of data in the eurIPFreg was maintained through internal plausibility checks, in which different items were put into a logical context, causing the generation of queries in case inconsistent entries were noted (e.g., if physician’s and patient’s report were not consistent with regard to signs of underlying collagen/vascular disease). These queries were addressed by the respective site investigator, asking for clarification of the issue.

All statistical procedures were performed using SPSS 24 (SPSS, IBM Corp). For baseline data, the summary descriptive statistic was generated with categorical data displayed as absolute numbers and relative frequencies. Continuous data are shown as mean (SD) for normally distributed data or as median (interquartile range) for nonparametric data. Comparisons between groups were performed using a *t*-test, Mann–Whitney U test, or a linear regression analysis. Correlation coefficient was determined by Spearman. The Chi²-test was used for nominally distributed characteristics. The results with a *p* < 0.05 were reported as statistically significant. The results were evaluated with the program GraphPad Prism 5 (GraphPad software, La Jolla, San Diego, CA, USA).

## 3. Results

### 3.1. Disease-Specific FeNO Distribution and Correlation with Pulmonary Function Test (PFT) Values

In total, FeNO measurements could be performed in the 94 patients of different groups. The demographics data together with clinical and pulmonary function (PFT) parameters are outlined in Table 1. The characteristic of ILD subgroups is displayed in Table S1 of the Supplementary file.

**Figure 1:**
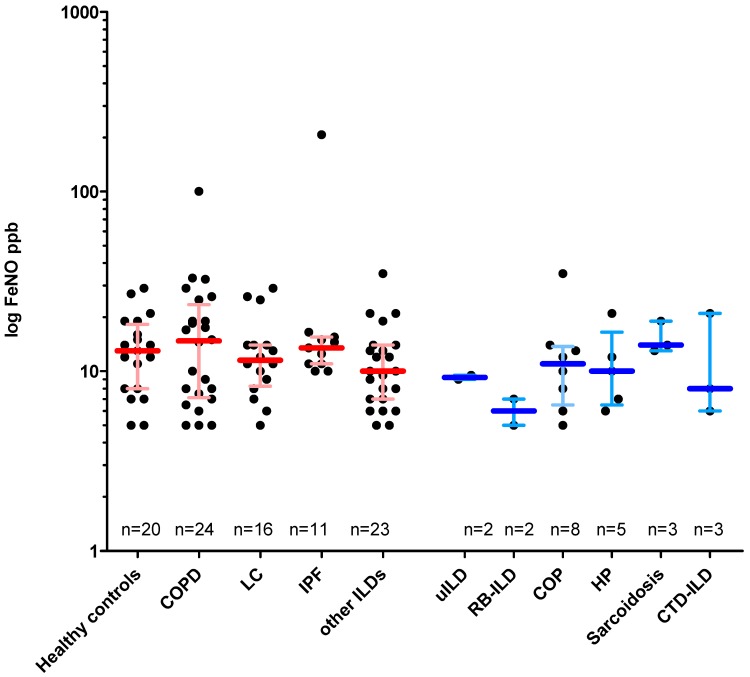
FeNO values in the different lung disease cohorts vs. healthy controls. Given are the median (horizontal bar) with interquartile range as well as single data (dots). uILD, RB–ILD, COP, HP, sarcoidosis and CTD–ILD have been summarized as “ILD” and are shown separately in blue on the right margin. Abbreviations: IPF: idiopathic pulmonary fibrosis, ILD: interstitial lung diseases, HP: hypersensitivity pneumonitis, COPD: chronic obstructive pulmonary disease, COP: cryptogenic organizing pneumonia, CTD–ILD: connective tissue disease-associated ILD, LC: lung cancer, NSIP: nonspecific interstitial pneumonia, RB–ILD: respiratory bronchiolitis ILD, uILD: unclassifiable ILD. If data not available from all patients, the number(s) is specified.

All groups showed a similar range of FeNO values (Figure 1). In order to assess the extent to which FeNO values relate with the clinical progression of pulmonary fibrosis, FeNO values for IPF and other ILDs were correlated with PFT values, blood gas analysis, 6MWD and spiroergometry in a linear regression analysis. In IPF, FeNO values were not significantly correlated to PFT values (Supplementary Figure S1a), with the exception between transfer coefficient for carbon monoxide (KCO) and FeNO of uncertain relevance (most probably due to one outlier). Likewise, the total ILD group including IPF showed no significant correlation of lung function values and FeNO as well. However, an inverse, significant correlation between FeNO and intrathoracic gas volume (ITGV) (r = −0.44, *p* = 0.03) as well as an inverse, highly significant correlation between FeNO and residual volume/total lung capacity RV/TLC (r = −0.69, *p* = 0.0003) were observed. The data are shown in Supplementary Figure S1b.

To further analyze the relationship between exercise capacity and FeNO in IPF, the values were correlated with the walking distance of the six-minute walk test (6MWD) and the VO2max values of the spiroergometry. There was no relevant correlation of the FeNO values with the 6MWD, either when restricted to IPF patients alone (Supplementary Figure S2, upper left panel) or when analyzed for all ILD patients including IPF (Supplementary Figure S2, upper right panel). The maximum oxygen uptake (VO2max) in spiroergometry showed a positive although insignificant correlation to the FeNO values for the IPF (r = 0.6) as well as for the total ILD cohort (r = 0.02) (Supplementary Figure S2), possibly related to one outlier.

### 3.2. Impact of Smoking Status and Medication on FeNO Measurements

To clarify the extent to which smoking behavior and drug intake influence the FeNO values, the FeNO values for all patients were analyzed in dependency of the following subgroups: Smoker status (never, active, former), as well as intake of medication (non-steroidal anti-inflammatory drugs (NSAID), steroids and proton pump inhibitors (PPI)).

To this end, actively smoking patients showed significantly lower FeNO levels than former smokers and never smoked patients (Supplementary Figure S3). The median FeNO values observed in patients with intake of NSAID, steroids or PPI were not significantly different than in those without the above-mentioned medication.

### 3.3. Intraindividual FeNO Variability and FeNO Values in Exacerbation

The intraindividual variability of FeNO was determined by repeated analyses (from 1 to 11 days between the measurements) in the IPF and COPD cohorts (Supplementary Figure S4). Overall, a good reproducibility of the FeNO measurements was shown.

To evaluate the impact of exacerbation on FeNO, we analyzed FeNo values of the whole cohort (Supplementary Figure S5) as well as of COPD patients (Supplementary Figure S6) with regard to the following exacerbation criteria: Formal diagnosis of exacerbation in the patient file, colored sputum, intake of antibiotics, proof of causative pathogen in sputum or bronchial suction samples. However, none of these criteria was associated with a meaningful difference in measured FeNO values.

### 3.4. Free and Total 8-Isoprostane Values in the EBC and the BALF

**Figure 2:**
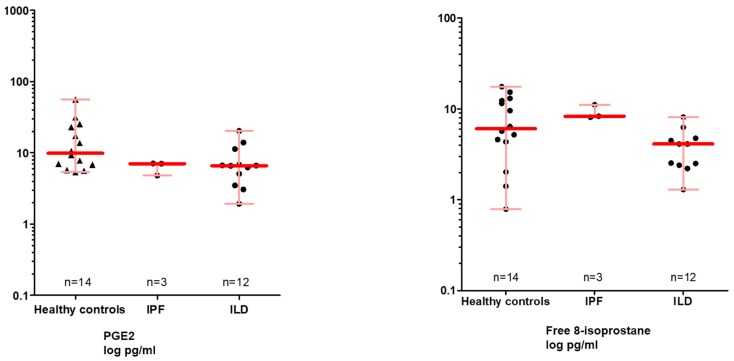
Free 8-isoprostane and PGE 2 in the EBC. Given is the median with interquartile range. HP, uILD, DIP, COP, sarcoidosis as well as CTD-ILD have been summarized as ILDs. Abbreviations: IPF—idiopathic pulmonary fibrosis, ILD—interstitial lung diseases. EBC was analyzed after concentrating its content. If data not available from all patients, the number (s) is specified.

The free 8-isoprostane in the exhaled breath condensate (EBC) was measured with a commercial ELISA (Cayman). In general, when analyzing EBC directly, the values were in the lower range of the standard curve and in a number of cases even below that. We therefore analyzed EBC after concentrating its content. In Figure 2 (right panel) it is shown that for those samples which were measurable after concentration, no significant difference was visible between IPF, other ILDs and healthy controls.

**Figure 3:**
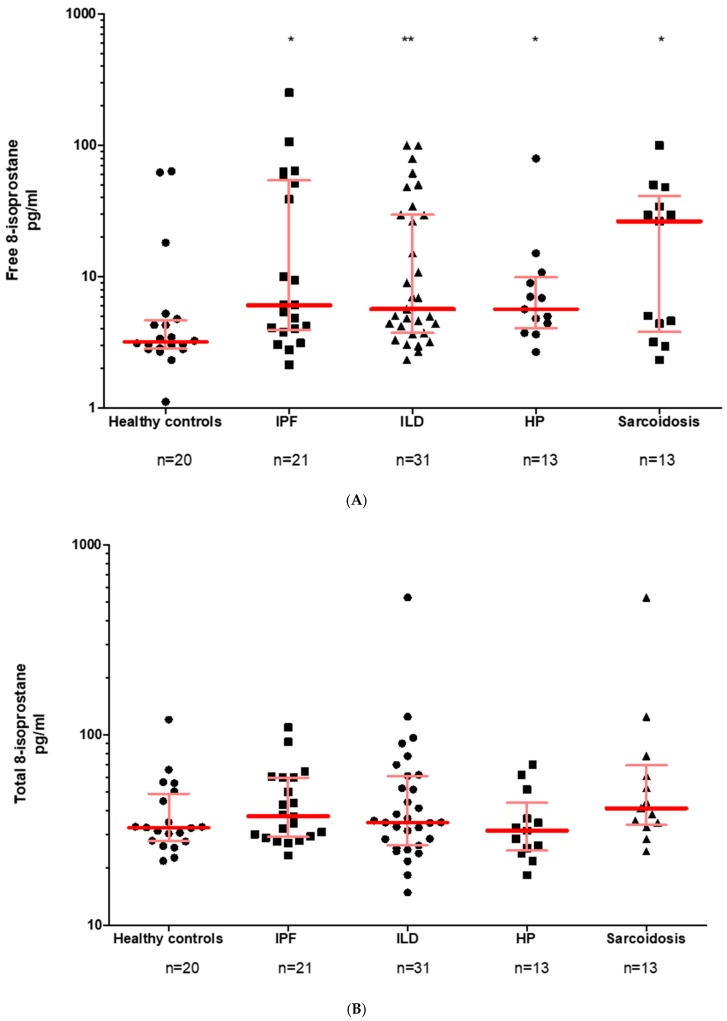

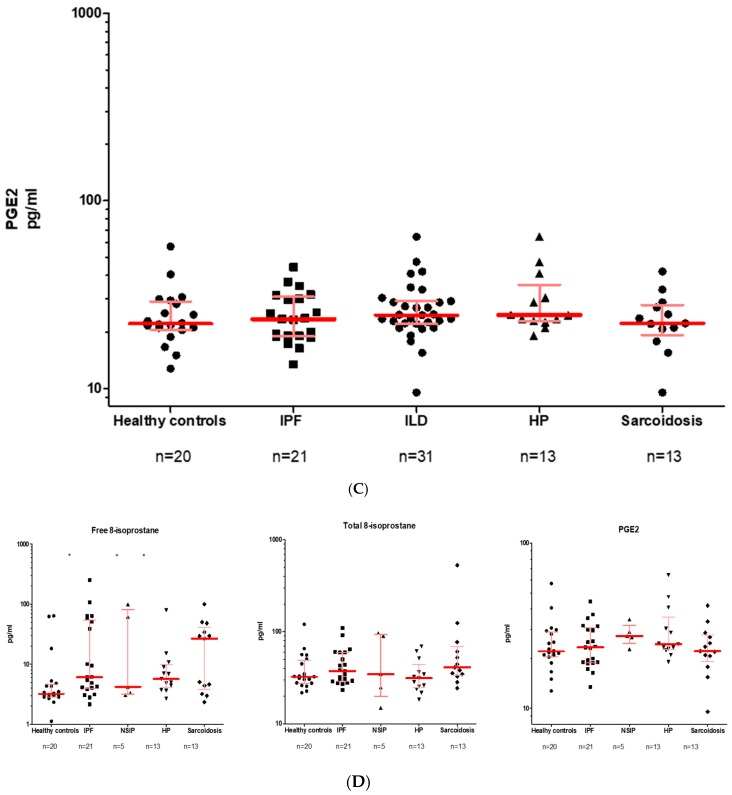
Distribution of PGE2 and 8-isoprostane in BALF. Data are given as median with interquartile range (**A**) Free 8-isoprostane (**B**) Total 8-isoprostane (**C**) PGE2 (**D**) ILD subgroup analysis in regard to all three above mentioned biomarkers. Abbreviations: IPF: idiopathic pulmonary fibrosis, ILD: interstitial lung diseases, NSIP: nonspecific idiopathic pneumonia, HP: hypersensitivity pneumonitis. If data not available from all patients, the number(s) is specified.

In BALF, the free 8-isoprostane, but not total 8-isoprostane, values were found to be significantly higher in IPF, HP and sarcoidosis as compared to healthy controls (Figure 3A,B).

### 3.5. PGE2 in the EBC and BALF

In the EBC, PGE2 was difficult to detect and each single patient sample yielded values below the smallest standard. Samples were therefore concentrated by lyophilization and again analyzed for PGE2 determination, all of which were measurable for PGE2 (Figure 2, left panel). Healthy controls showed the highest PGE2 median value (9.9 pg/mL), while the IPF (7.03 pg/mL) and the ILD (6.61 pg/mL) appeared to have slightly lower values. There was no significant difference between the groups.

In a complementary approach, we analyzed PGE2 in BALF, which was found to be detectable in all samples (see Figure 3C). Overall, the medians for healthy subjects (22.08 pg/mL), IPF patients (23.34 pg/mL) and ILD patients (24.51 pg/mL) were quite similar, so there was no significant difference between the groups. Looking more closely at the ILD subgroups, NSIP patients (27.33 pg/mL) showed the highest PGE2 values, followed by HP (24.51 pg/mL) and sarcoidosis (22.18 pg/mL) (Figure 3D). Again, there was no significant difference here either.

### 3.6. Eicosanoids Do Not Reflect Disease Progression

**Figure 4:**
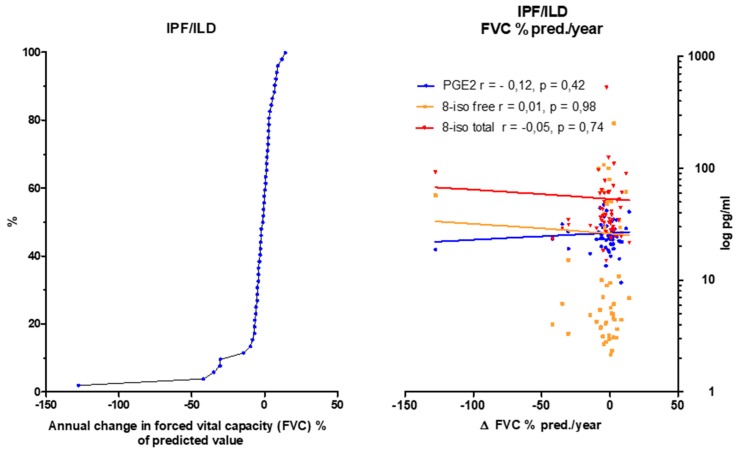
Correlation of the annual change in forced vital capacity (FVC; % of predicted value) in IPF and ILD patients and PGE2, total and free 8-isoprostane values in BALF. Left panel: The annual change of the FVC is summarized in the cumulative frequency diagram. Right panel: Correlation of FVC (% of predicted value) to PGE2 and 8-isoprostane. Abbreviations: FVC: forced vital capacity, PGE2: prostaglandine E2, r: correlation coefficient according to Spearman, 8-iso: 8-isoprostane. If data not available from all patients, the number(s) is specified.

**Figure 5:**
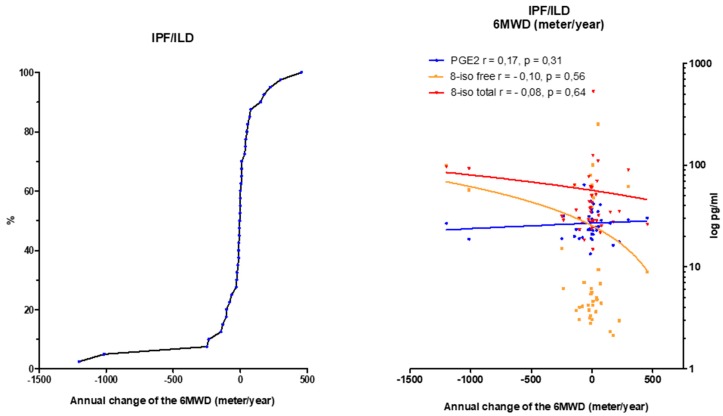
Correlation of the annual change in 6MWD (meter/year) in IPF and ILD patients and PGE2, total and free 8-isoprostane values in BALF. Left panel: The annual change of the 6MWD is summarized in the cumulative frequency diagram. Right panel: Correlation of 6MWD to PGE2 and 8-isoprostane. Abbreviations: 6MWD: walking distance in six minutes, PGE2: prostaglandine E2, r: correlation coefficient according to Spearman, 8-iso: 8-isoprostane. If data was not available from all patients, the number(s) is specified.

In order to assess the extent to which 8-isoprostane and PGE2 levels from BALF samples reflect the disease progression, the annual change in FVC (Figure 4), DLco (Supplementary Figure S7) and 6MWD (Figure 5) was correlated with PGE2 and 8-isoprostane values in BALF by a linear regression analysis. For this evaluation IPF and ILD were taken as one group. Unfortunately, there were no significant correlations between each eicosanoid and the course of FVC, DLco or 6 MWD over time.

## 4. Discussion

The aim of this work was to investigate if FeNO or exhaled eisocanoids could be of diagnostic help in ILD and other chronic lung diseases. We also performed correlation analyses of FeNO, PGE2 or 8-isoprostane values and PFT parameters to investigate the prognostic relevance of these markers and their relation to medication.

Unfortunately, we did not observe any meaningful or significant differences between FeNO or eicosanoid values of the different cohorts as well as controls. Yet, for reasons not understood, some patients showed highly elevated FeNO values. Re-evaluation of these cases did not forward any particular explanation (e.g., exacerbation). In general, it was neither possible to differentiate between the kind of disease, to detect exacerbation or to correlate FeNO or eicosanoid values with lung function parameters DLco or 6MWD. In addition, analysis of arachidonic acid derivates in EBC was challenging (PEG2) or impossible (8-isoprostane), whereas it worked out well in BALF. Some group-specific, significant differences were observed with regard to free 8-isoprostane in BALF, the meaning of which appears questionable in light of the unchanged total 8-isoprostane values and the missing correlation to progression of disease.

Previous studies have shown that measurement of FeNO appears to be a quantitative, non-invasive, simple and safe method of measuring airway inflammation and to assess activity of airway disease, especially in asthma [30]. In our study, FeNO measurement and result interpretation were done according to an official ATS guideline [31].

In our study, a good reproducibility of FeNO results has been proven, which is in line with other studies, where FeNO measurements demonstrated high reproducibility and low daily variability in healthy and asthmatic children and adults [32]. We did not have asthma patients in our cohort, but determined the FeNO values in 20 healthy controls. With a median of 13 ppb (IQB 8–18.25), the distribution corresponded to previous observations, according to which FeNO values of healthy persons range from 4–20 ppb [33].

When interpreting FeNO measurements, individual factors should always be considered. In adults, a positive correlation was observed between the FeNO levels and age of the patients [34]. We also detected decreased FeNO values in active smokers, which is in line with previous reports; e.g., Lu at al compared FeNO levels between ex-smokers and current smokers in COPD and reported that FeNO levels in ex-smokers were higher than those in current smokers [35].

The effect of steroids on the FeNO values had been discussed differently in the literature and seems to depend on the underlying disease and its pathomechanism [22]. Lehtimäki et al. reported the FeNO values of IPF and HP patients appear to be responsive to steroids [36]. However, no significant change in steroid intake across all groups was observed in this work. Our study showed that steroid-negative COPD patients display slightly higher FeNO values than those treated with steroids.

With regard to changes in FeNO in IPF, Saleh et al. showed that NO values might be upregulated in IPF patients, with a significant increase in early to intermediate stage of the disease, suggestive of a valuable role of constitutively produced NO in pulmonary homeostasis and of a protective effect on lung architecture [37]. In his study, IPF patients showed strong expression of nitrotyrosine and NOS was seen in macrophages, neutrophils, and alveolar epithelium, especially in the early to intermediate stage of IPF. The active stage of IPF was associated with increased inflammatory and alveolar expression of nitrotyrosine and NOS, where increased production of NO and peroxynitrite seemed to contribute to the oxidative damage [37].

Paredi et al. showed elevated FeNO values in IPF patients (MW with SD: 11.2 ± 1 ppb) compared to healthy non-smokers (6.9 ± 0.5 ppb) (flow rate: 5 L/min = 83.3 mL/s) [38]. The FeNO values in our IPF patients were comparable to those of Paredi, but were only slightly elevated as compared to controls. In contrast, Guilleminault et al. measured a median FeNO value of 22 ppb (IQB: 17–30 ppb, flow rate: 50 mL/s), which is significantly higher than the IPF patients researched here [19]. This may be due to differences in the device (Belgium Hypair FeNO, Medisoft). Such view is also supported by the fact that in Guilleminault’s report all FeNO values were reported to be higher as compared to our study (IPF: 22 ppb, chronic EAA: 51 ppb, drug-induced fibrosis: 19 ppb, CTD–ILD: 25 ppb).

In another study, Cameli et al. compared FeNO values in idiopathic interstitial pneumonitis (IIP) patients (22 IPF, 8 NSIP) and healthy controls (*n*: 30) at different flow rates. At 50 mL/s IPF patients showed similarly high levels (22.3 ± 8.4 ppb; MW ± SD) as those reported by Guilleminault, but were lower as compared to Schildge et al. (27.6 ± 16.3 ppb; MW ± SD) [39,40]. Again, FeNO measurements in the Cameli and the Guilleminault studies were performed with the same device (Hypair FeNO, Medisoft). Absolute FeNO values have been reported to depend on the underlying method of NO measurement of the device. To this end, lower values of FeNO have been noted when using the chemiluminescence method (NIOX Mino, Aerocrine) versus the electrochemical method (Hypair FeNO, Medisoft) [29].

With regard to the eisocanoid measurements, it had been previously suggested that PGE2 is down-regulated in IPF patients, thus favoring the development of pulmonary fibrosis [41,42]. In IPF, reduction in PGE2 level is assumed to result from a defective COX2 pathway [43]. Despite the many references in the literature, no significant PGE2-level difference between the IPF, ILD and the healthy controls could be found in BALF in our study. Compared to the other ILD subgroups, IPF did not appear to have the lowest PGE2 values in the EBC.

Free and total 8-isoprostane are markers for oxidative stress-induced lipid peroxidation. Elevated levels of free 8-isoprostane have been used as markers of oxidative stress in asthma, COPD, pulmonary sarcoidosis, ILD and IPF [30,44]. Montuschi et al. described elevated 8-isoprostane values in the BALF of IPF (47.7 ± 7 pg/mL) versus healthy subjects (9.6 ± 0.8 pg/mL) [45]. Our mean value of free 8-isoprostane of 31.07 pg/mL in BALF in IPF is not that much different as compared to theirs, but our healthy subjects showed clearly higher 8-isoprostane values.

One of our study limitations was the sample size in the ILD subgroup analysis. In addition, we reported the results of the consecutive patients without withdrawal of any medication, thus it might not reflect the natural course of the lung diseases. Especially in the pirfenidone group, one could assume the possible influence this medication has on the FeNO levels. Further studies, taking into account a larger number of patients and longer follow up periods, are recommended to deeper evaluate the role of biomarkers in diagnostic and prognosis of interstitial lung diseases.

## 5. Conclusions

In summary, no significant and meaningful difference in the FeNO, PGE2, or 8-isoprostane values in EBC or BALF could be observed between different forms of ILD’s, especially IPF, and other lung diseases such as COPD or LC and healthy controls. In addition, we failed to observe a relevant correlation of these values with lung function, gas exchange or exercise parameters or with exacerbation or treatment status.

Therefore, our results do not support previous studies in IPF patients and other ILDs, according to which NO is an important marker for the assessment of disease severity. To our understanding, FeNO or PGE2 or 8-isoprostane measurements in BALF do not offer as diagnostic or prognostic markers, nor do they indicate exacerbation.

## Figures and Tables

**Table 1:** Demographics and clinical and functional parameters in the IPF, ILD, COPD and LC cohort (FeNO measurements).

Parameters	Healthy Controls	IPF	ILD	COPD	Lung Cancer	Pooled Analysis	Test
	*n* = 20	*n* = 11	*n* = 23	*n* = 24	*n* = 16	*p* Value	
Age	30 (25–49)	70 (66–73) ***	68 (49–73) ***	68 (61–74)	65 (58–70)	<0.0001	KW
Gender (female/male)	19/1	2/9	10/13	10/14	3/13	<0.0001	KW
Active smoker, *n* (%)	8 (40)	1 (9)	2 (9)	4 (17)	2 (13)	<0.0001	KW
Never smoked, *n* (%)	10 (50)	3 (27)	8 (35)	4 (17)	0 (0)		
Ex smoker, *n* (%)	2 (10)	7 (64)	13 (57)	16 (67)	14 (88)		
CRP (mg/dl)		0.5 (0.3–1.3)	0.5 (0.2–1.3)	0.7 (0.43–1.4)	0.5 (0.15–1.08)	0.29	KW
VC % pred.	102 (87–112)	75 (47–83) ***	77 (55–92)	68 (46–81), *n* = 23	79 (66–87)	<0.0001	KW
FVC % pred.	97 (83–110)	68 (45–88)	83 (47–97) **	57 (40–72), *n* = 22 ***	76 (62–87)	<0.0001	KW
TLC % pred.	103 (94–108)	57 (47–75) ***	78 (62–98) **	87 (78–119), *n* = 22	85 (79–97)	<0.0001	KW
FEV1 % pred.	95 (87–109)	90 (57–95)	84 (57–109) **	53 (43–66), *n* = 23 ***	78 (67–91)	<0.0001	KW
FEV1/VC % pred.	99 (91–106)	112 (109–138)	113 (104–131) ***	83 (68–104)	106 (92–110)	0.0003	KW
ITGV % pred.	99 (91–112)	64 (50–90) **	88 (66–109)	113 (93–170), *n* = 23	91 (77–114)	0.0015	KW
RV % pred.	104 (91–124)	50 (24–80) ***	89 (57–121) *	140 (86–197), *n* = 22	98 (84–133)	0.0012	KW
RV/TLC % pred.	104 (93–112)	60 (50–142) **	110 (78–124) *	147 (120–172), *n* = 23 **	112 (95–137)	0.0008	KW
R tot kPa x s/l	0.23 (0.21–0.29)	0.32 (0.25–0.65)	0.34 (0.26–0.43) ***	0.61 (0.5–0.8), *n* = 23 ***	0.43 (0.33–0.54) *	<0.0001	KW
DLCO % pred.	91 (79–108)	38 (26–54), *n* = 10 ***	57 (37–74), *n* = 17 *	20 (16–31), *n* = 7 ***	40 (31–52), *n* = 6 *	<0.0001	KW
KCO % pred.	88 (77–99)	53 (33–62), *n* = 10**	67 (65–93), *n* = 15		22.5 (13–32), *n* = 2 *	0.0003	KW
SaO_2_ in %	97 (95–98), *n* =10	94 (94–95)	96 (95–97)*	94 (92–95) **	95 (94–96)	0.0048	KW
pO_2_ (mmHg)	80 (71–89), *n* =10	73 (69–75)	81 (74–87)	69 (63–76.5)	75.5 (69–83)	0.0952	KW
pCO_2_ (mmHg)	34 (33–37), *n* =10	41 (36–46)	39 (37–42)	42 (40–48.25)	39 (37–44.25)	0.0147	KW
LTOT, *n* (%)	0 (0)	3 (27)	3 (15)	8 (33.3)	2 (12.5)	0.0416	Chi²
6 MWD (meters)		270 (120–360)	360 (293–480), *n* = 10	360 (270–360), *n* = 4	300, *n* = 1	0.1557	KW
VO_2_max (ml/kg/min)		13 (11.5–21.5), *n* = 5	19 (14–25), *n* = 7	17, *n* = 1	18 (14–22), *n* = 2	0.6404	KW
NSAID, *n* (%)	0 (0)	6 (55)	9 (39) **	8 (33,3)	8 (50)	0.005	KW
PPI, *n* (%)	0 (0)	5 (45)	10 (43)	14 (58) ***	12 (75)	<0.0001	KW
Systemic steroids	0 (0)	8 (73)	15 (65)	19 (79) ***	7 (44)	<0.0001	KW
Pirfenidone, *n* (%)	0 (0)	5 (45)	0 (0)	0 (0)	0 (0)		

The data are given in median (interquartile range), numbers or percentage (*n*, %). ILDs include the uILD, RB–ILD, COP, HP, sarcoidosis, and CTD–ILDs. Calculation of *p* values was performed with use of Kruskal–Wallis (KW) Test, Dunnett’s multiple comparison tests and One–way Anova; Chi²–Test (Chi²) was used for nominal variables, and Mann–Whitney–U (MWU) Test for BAL. * *p* < 0.05, ** *p* < 0.01, *** *p* < 0.0001. The ILD cohort is further characterized in Table 1 of the supplementary file. If data was not available from all patients, the number(s) is specified. LC: lung cancer, IPF: idiopathic pulmonary fibrosis, ILD: interstitial lung diseases, COPD: chronic obstructive pulmonary disease, LC: lung cancer, uILD: unclassifiable ILD, RB-ILD: respiratory bronchiolitis associated ILD, COP: cryptogenic organizing pneumonia, COPD: chronic obstructive pulmonary disease, HP: hypersensitivity pneumonitis; CTD-ILD: connective tissue disease-associated ILD.

## Abbreviations

Idiopathic pulmonary fibrosis (IPF), European IPF Registry (eurIPFreg), European IPF Biobank (eurIPFbank), Interstitial lung diseases (ILD), Chronic Obstructive Pulmonary Disease (COPD), High resolution computed tomography (HRCT), Blood gas analysis (BGA), Bronchoalveolar lavage fluid (BALF), Long term oxygen treatment (LTOT), Forced expiratory volume (FEV1), Residual volume (RV), Total lung capacity (TLC), Vital capacity (VC), Forced vital capacity (FVC), Diffusing capacity of the lung for carbon monoxide (DLC0), Partial pressure of oxygen (pO2), Partial pressure of carbon dioxide (pCO2), Lung cancer (LC), alveolar NO concentration (CaNO), Cryptogenic Organizing Pneumonia (COP), exhaled breath condensate (EBC), Enzyme Linked Immunosorbent Assay (ELISA), fractionated exhaled nitrogen monoxide (FeNO), matrix metalloproteinase (MMP), non-steroidal anti-inflammatory drugs (NSAID), Non-specific Interstitial Pneumonia (NSIP), prostaglandin E 2 (PGE2), Pulmonary hypertension (PH), proton pump inhibitors (PPI), Respiratory Bronchiolitis-associated Interstitial Lung Disease (RB–ILD), oxygen saturation (SaO2), Universities of Giessen and Marburg Lung Center (UGMLC), vital capacity (VC), six-minute walking distance (6 MWD).

## Supplementary Materials

The following are available online at https://www.mdpi.com/2077-0383/8/5/643/s1.
