# Supplementary material for: Exhalative Breath Markers Do Not Offer for Diagnosis of Interstitial Lung Diseases: Data from the European IPF Registry (eurIPFreg) and Biobank
Source: J Clin Med. 2019 May 9;8(5):643. doi: 10.3390/jcm8050643 (PMC6572439; doi:10.3390/jcm8050643)
Supplement: Supplementary file 1 [file jcm-08-00643-s001.zip › Supplementary Figures.pdf]

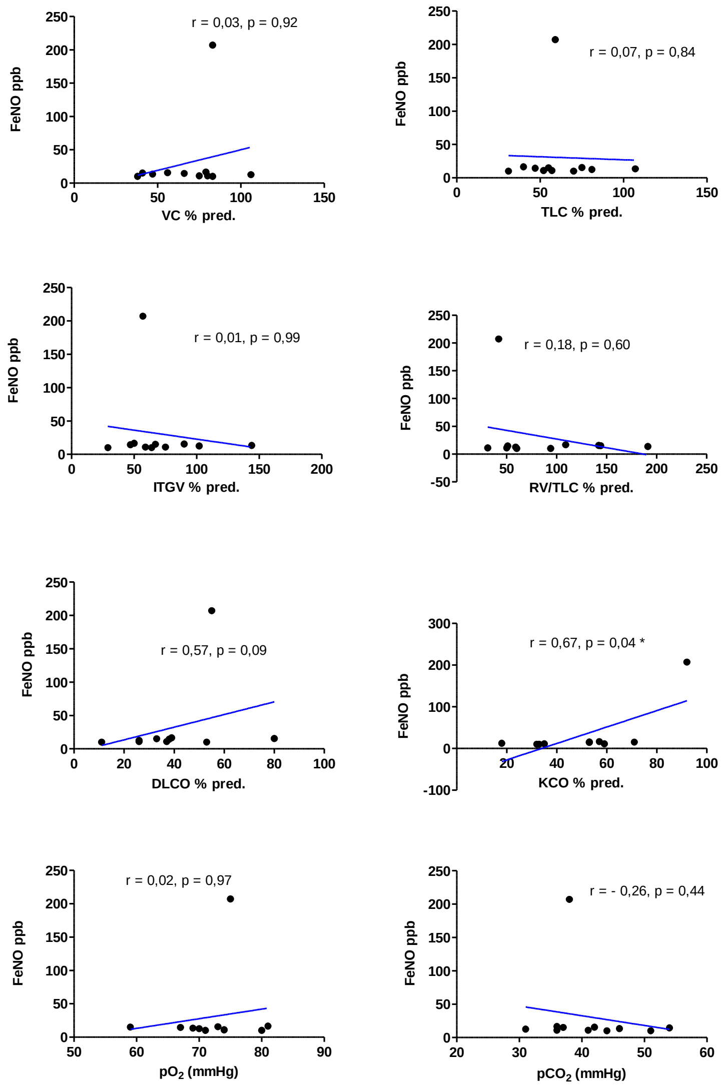

Suppl. Figure 1a. Correlation of lung function with FeNO in IPF patients.

Abbreviations: VC = vital capacity, TLC = total lung capacity, ITGV = intrathoracic gas volume, RV / TLC = relative hyperinflation, DLCO = diffusion capacity for CO, KCO = alveolar volume corrected DLCO, pO2 = oxygen partial pressure, pCO2 = partial pressure of carbon dioxide. r = correlation coefficient according to Spearman.

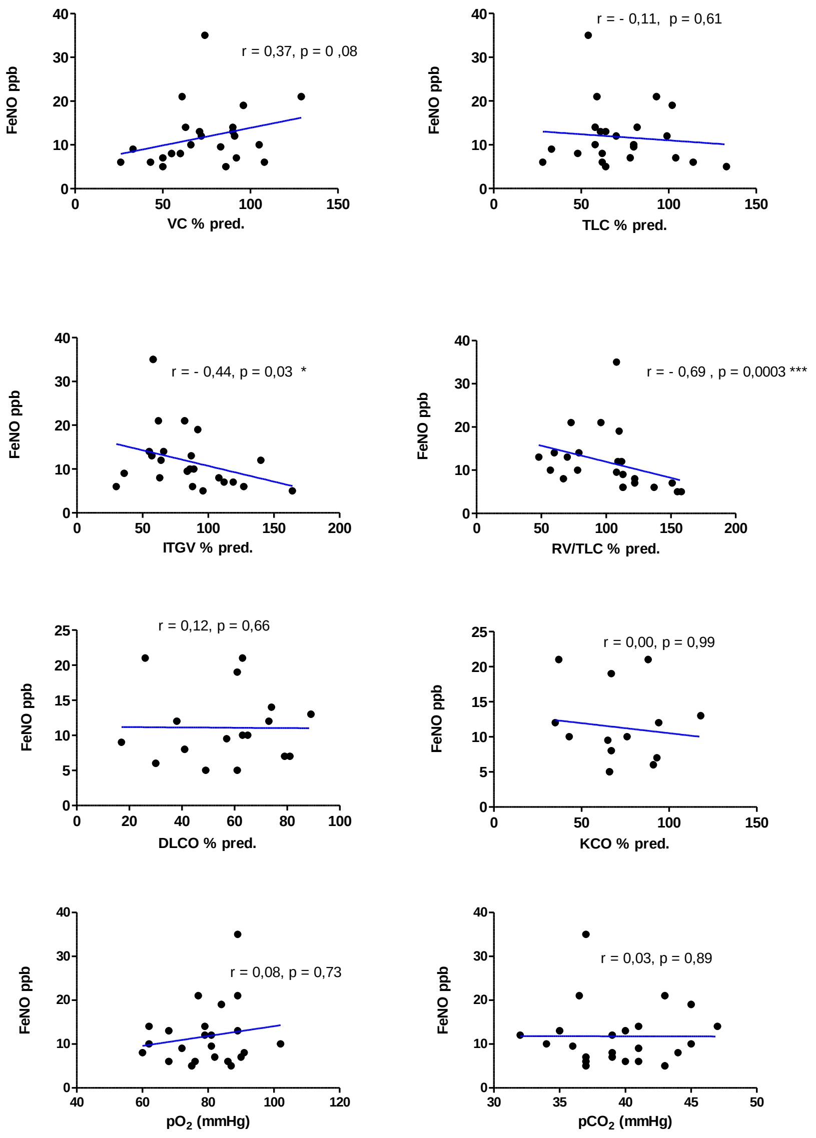

Suppl. Figure 1b. Correlation of lung function and DLco with FeNO in ILD patients. Abbreviations: Linear regression analysis of lung function parameters with FeNO values in the IPF cohort. VC = vital capacity, TLC = total lung capacity, ITGV = intrathoracic gas volume, RV / TLC = relative hyperinflation, DLCO = diffusion capacity for CO, KCO = alveolar volume corrected DLCO, pO2 = oxygen partial pressure, pCO2 = partial pressure of carbon dioxide. r = correlation coefficient according to Spearman, p = level of statistical significance.

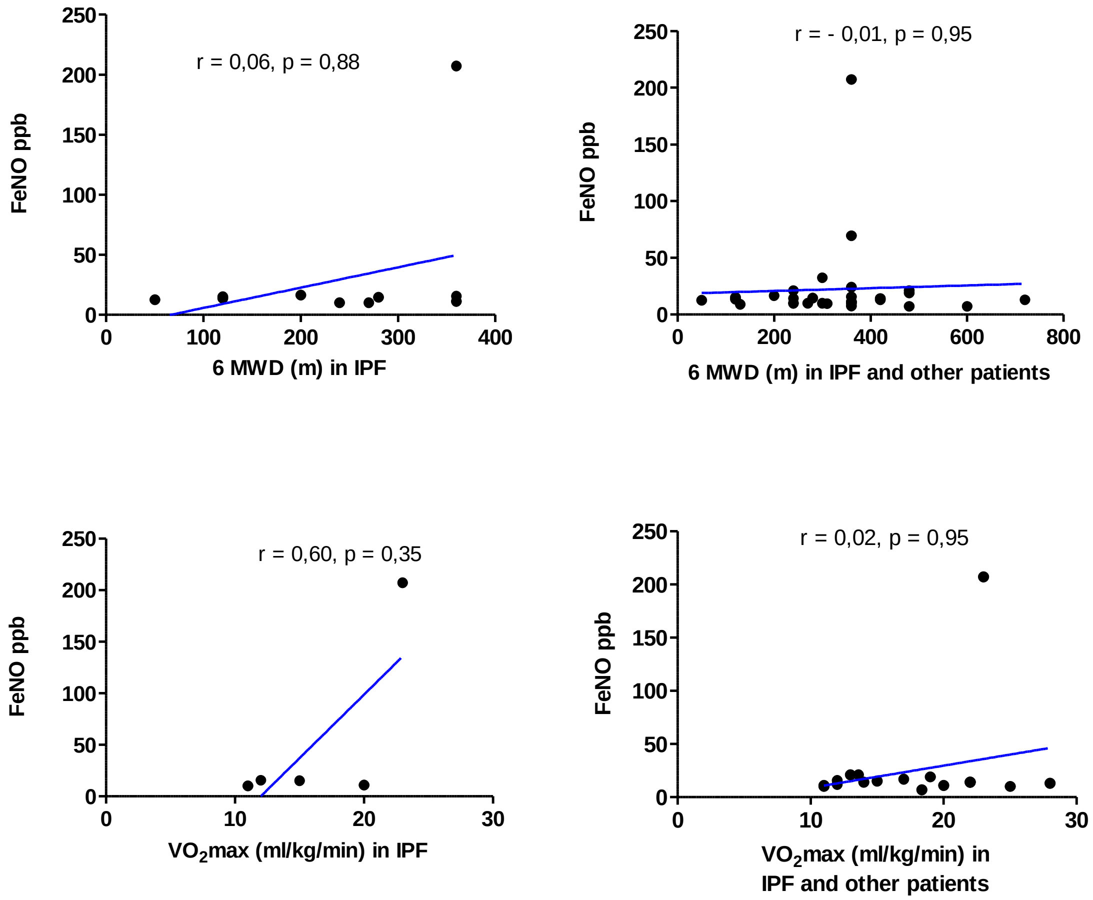

Suppl. Figure 2. Correlation of 6 MWD (m) and VO2max (ml/kg/min) in the spiroergometry to FeNO.

Abbreviations: 6 MWD = six minute walking distance test (m), VO2max = maximum oxygen uptake in spiroergometry, r = correlation coefficient according to Spearman, p = level of statistical significance.

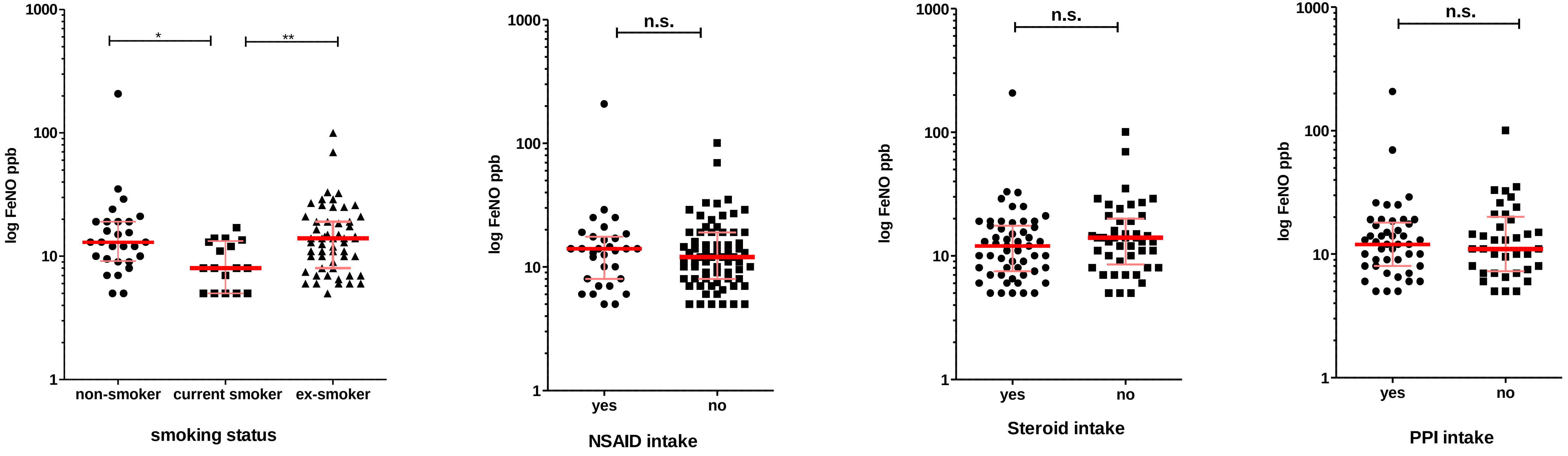

Suppl. Figure 3. Impact of smoking and co-medication on FeNO.

Abbreviations: For all subjects, FeNO is given in dependency of smoking status, steroids, NSAID and PPI intake. Shown are the median with interquartile range, p = level of statistical significance (Kruskal-Wallis test and Mann-Whitney test).

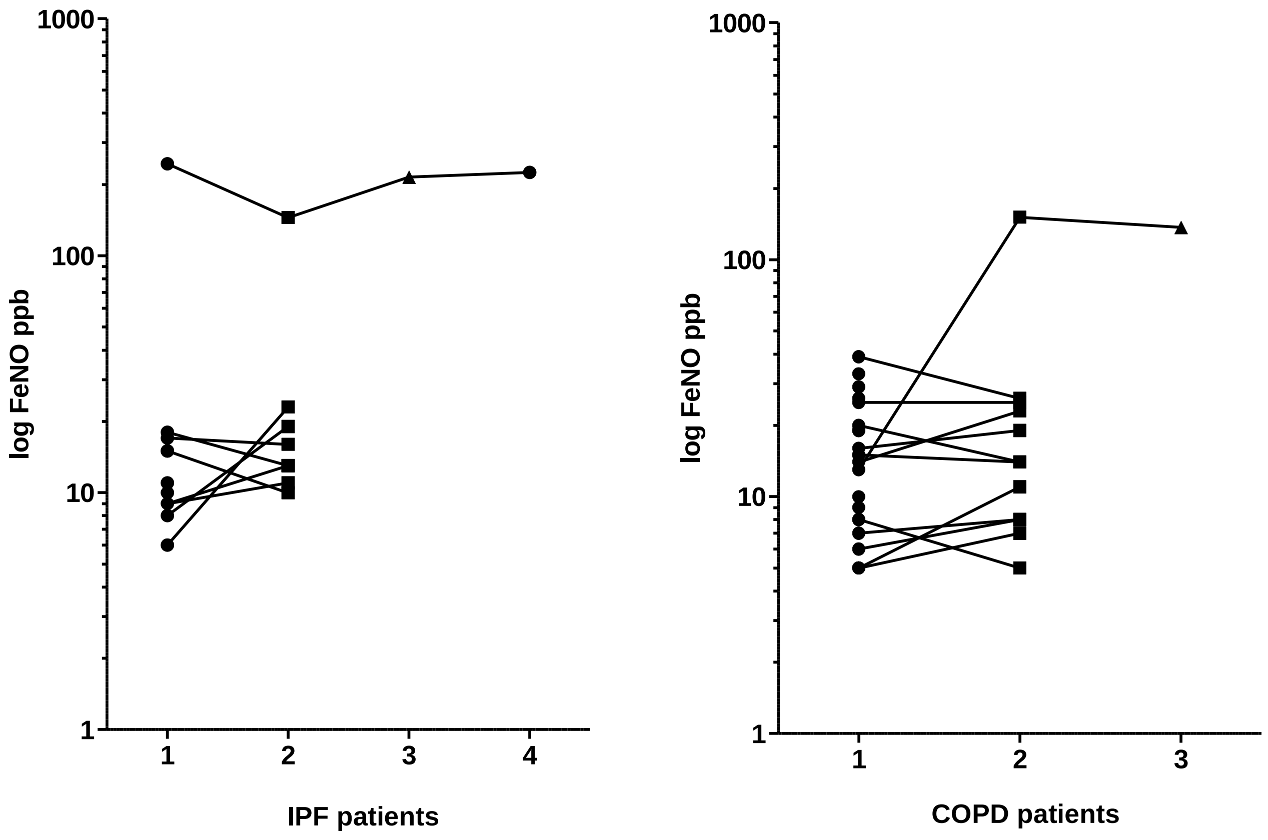

Suppl. Figure 4: Intraindividual variability of FeNO measurements. Abbreviations: Given are repeated FeNO measurements for the IPF (left) and COPD (right) patients within a period of max. eleven days. Each line represents one patient.

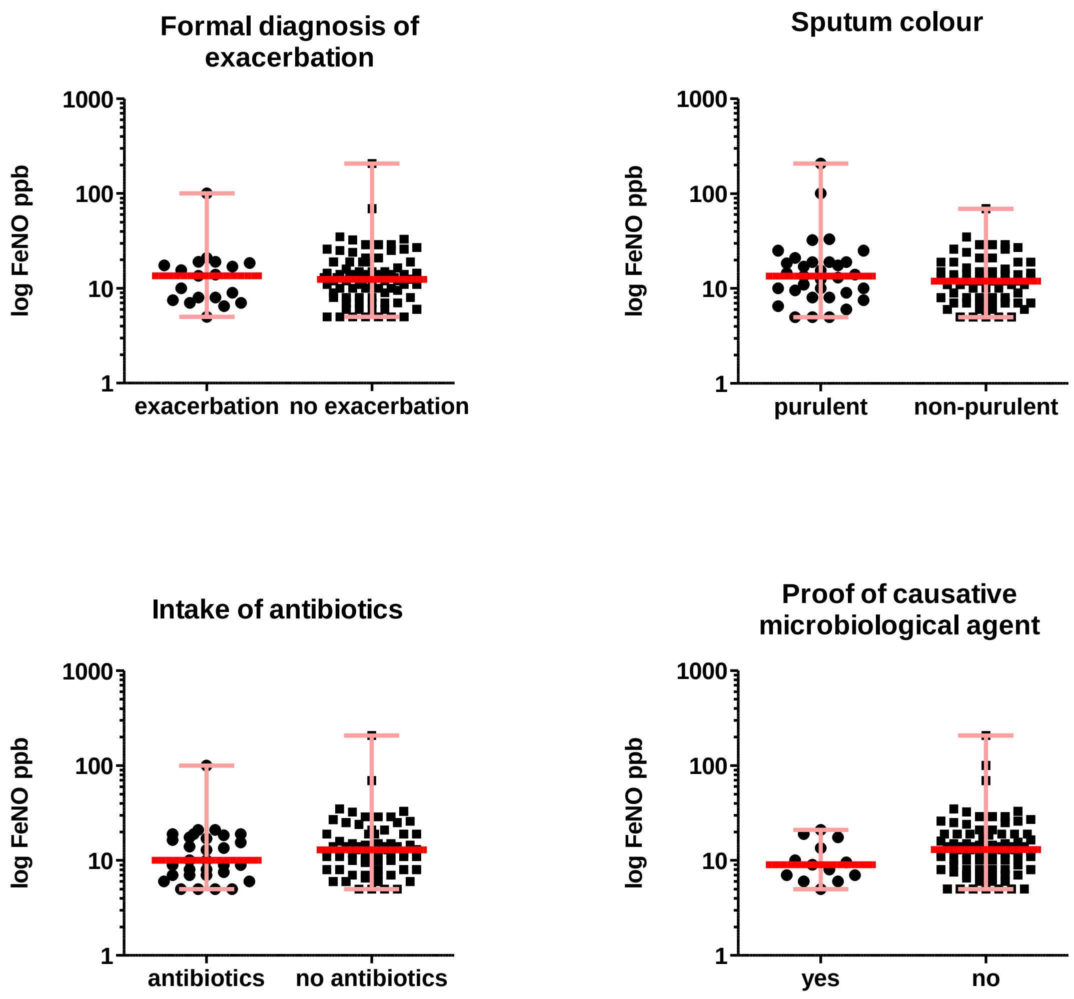

Suppl. Figure 5. Distribution of FeNO levels of all patients, stratified according to clinical parameters: Exacerbation, sputum discoloration, detection of causative pathogen in sputum and intake of antibiotics. Abbreviations: AB = antibiotics. Given is median values with interquartile range.

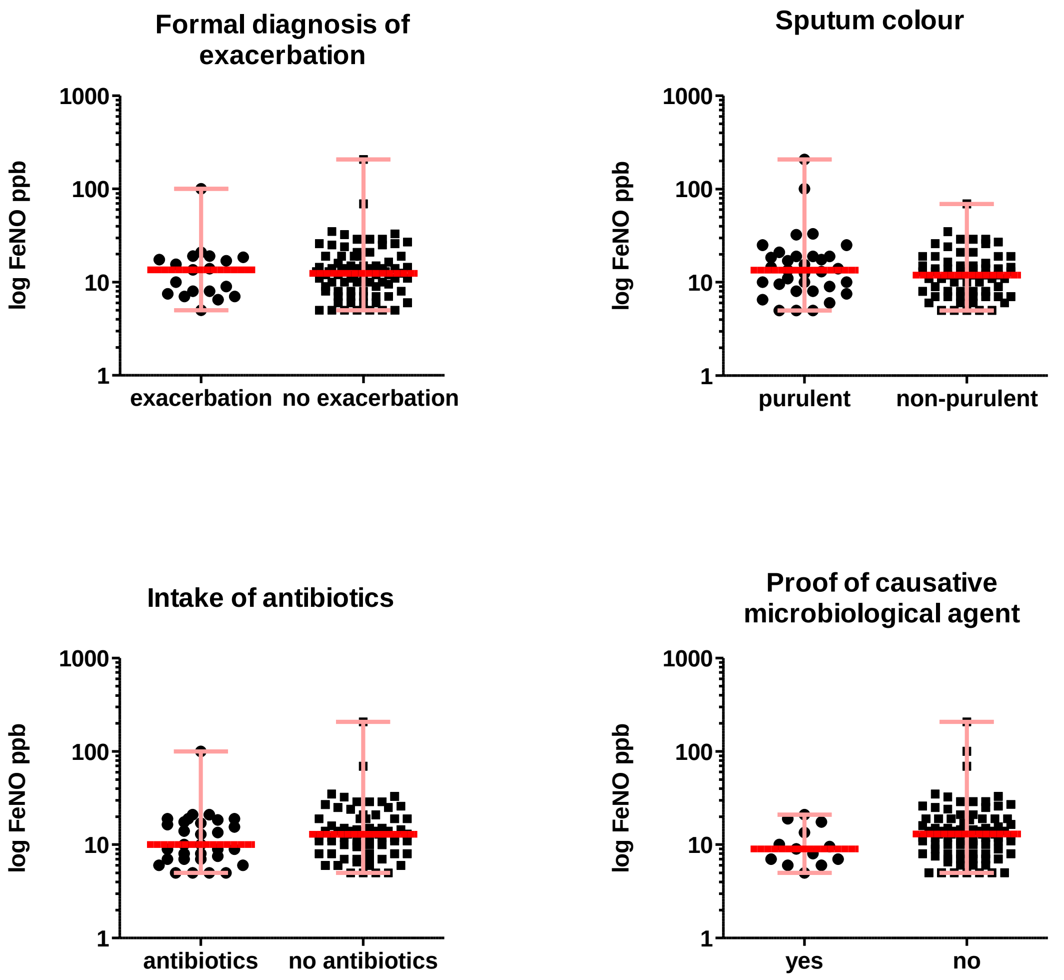

Supp.Figure 6. Correlation of FeNO values in COPD patients in dependency of the exacerbation criteria: Formal diagnosis of exacerbation in the patients file, proof of causative pathogen in respiratory tract, antibiotic and steroid intake. Median with interquartile range are given.

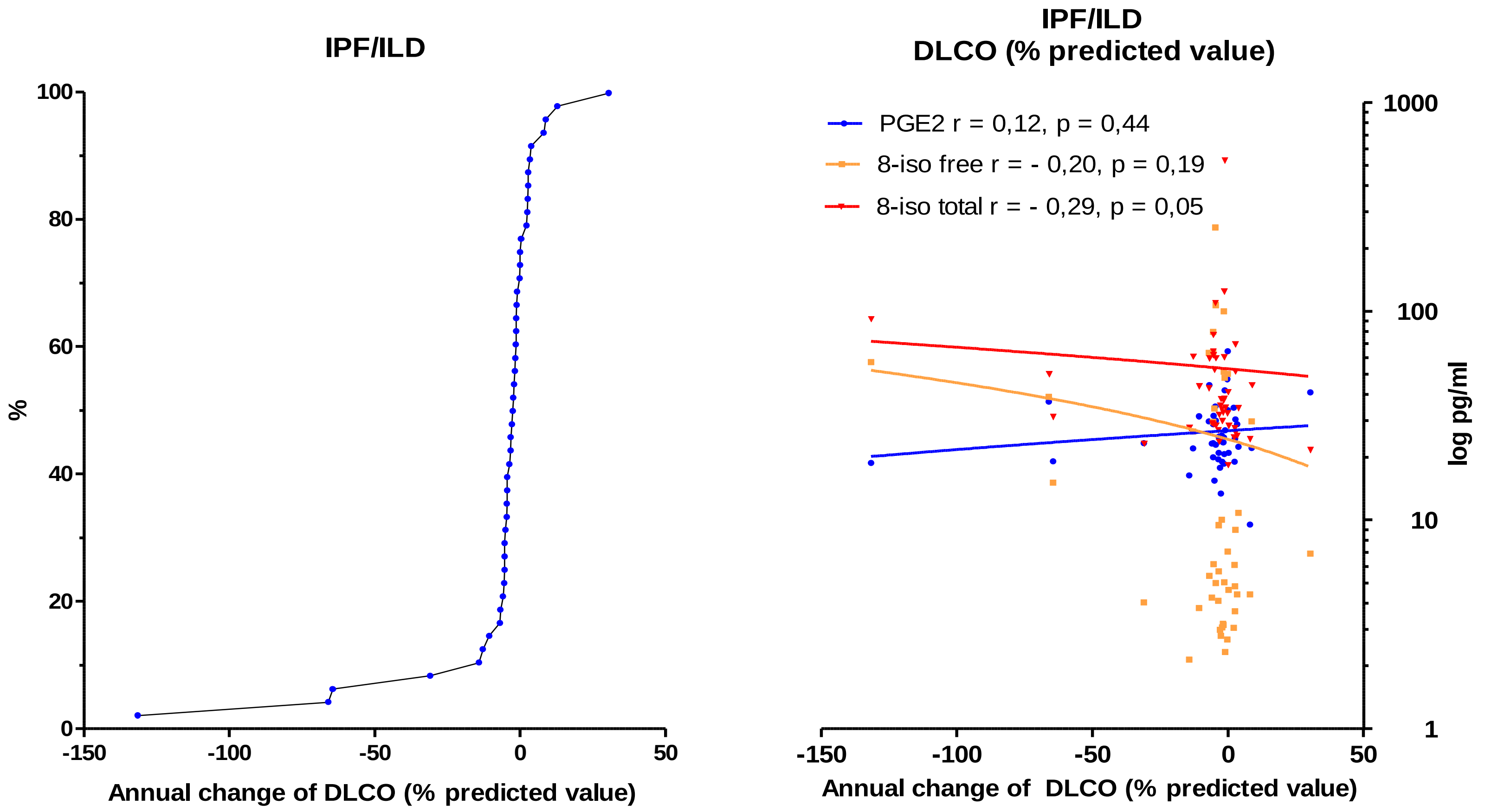

Suppl. Figure 7. Correlation of the annual change in diffusion capacity for carbon monoxide (DLCO; % of predicted value) in IPF and ILD patients and PGE2, total and free 8-isoprostane values in BALF.

Left panel: The annual change of the DLCO is summarized in the cumulative frequency diagram.

Right panel: Correlation of DLco (% of predicted value) to PGE2 and 8-isoprostane.

Abbreviations: DLCO -diffusion capacity for carbon monoxide , PGE2- prostaglandine E2, r -correlation coefficient according to Spearman, 8-iso- 8 isoprostane.
