# Supplementary material for: Exhalative Breath Markers Do Not Offer for Diagnosis of Interstitial Lung Diseases: Data from the European IPF Registry (eurIPFreg) and Biobank
Source: J Clin Med. 2019 May 9;8(5):643. doi: 10.3390/jcm8050643 (PMC6572439; doi:10.3390/jcm8050643)
Supplement: Supplementary file 1 [file jcm-08-00643-s001.zip › Supplementary Tables.pdf]

Journal of *Clinical Medicine*

MDPI

Supplementary Table S1. Demographics, clinical and lung function parameters in ILD subgroups (apart from IPF), FeNO measurements.

	uILD n = 2	RB-ILD n = 2	COP n = 8	HP n = 5	Sarcoidosis n = 3	CTD-ILD n = 3	Pooled analysis, p-value	Test
Age	75 (69-81)	39 (34-43)	71 (65-76)	69 (50-74)	50 (42-68)	62 (44-67)	0.1026	KW
Gender (♀/♂)	2/0	2/0	2/6	2/3	0/3	2/1	0.0977	Chi^2
Active smoker, n (%)	0 (0)	2 (100)	0 (0)	0 (0)	0 (0)	0 (0)		
Never smoked, n (%)	2 (100)	0 (0)	3 (38)	2 (40)	1 (33)	0 (0)	0.0014	Chi^2
Ex-smoker, n (%)	0 (0)	0 (0)	5 (63)	3 (60)	2 (67)	3 (100)		
CRP (mg/dl)	1.8 (0,3-3,3)	0.95 (0.7-1.2)	0.35 (0.23-2.03)	0.5 (0.15-2.75)	0.8 (0.3-1.2)	0.1 (0-22.6)	0.9348	KW
BAL: (ml)	70 (55-85)	80, n: 1	68 (56-72)	73 (65-84), n: 4	60 (55-65)	73 (73-73), n: 2	0.3243	MWU
Macrophages %	50 (45-55)	93, n: 1	78 (62-89)	51 (17-76), n: 4	72 (45-72)	71 (57-85), n: 2	0.2726	MWU
Neutrophils %	20 (2-37)	6, n: 1	4 (1-9)	4 (2-7), n: 4	11 (4-13)	20 (10-30), n: 2	0.3619	MWU
Eosinophils %	7.5 (7-8)	0, n: 1	1.5 (0-3.8)	2 (0.5-5.8), n: 4	0 (0-1)	1 (0-2), n: 2	0.2202	MWU
Lymphocytes %	23 (11-35)	1, n: 1	13 (6-26)	39 (18-79), n: 4	24 (16-42)	8 (3-13), n: 2	0.1631	MWU
CD4/CD8 ratio	5.8, n: 1		1.65 (0.45-3.85), n: 6	1.9 (0.65-2.63), n: 4	1.8 (1.8-7.5)	50, n: 1	0.2890	MWU
VC % pred.	62 (40-83)	64 (35-92)	87 (64-103)	61 (38-97)	71 (63-96)	74 (55-108)	0.8230	KW
FVC % pred.	67 (42-92)	61 (31-90)	83 (48-104)	65 (42-100)	83 (71-97)	83 (67-112)	0.8450	KW
TLC % pred.	72 (63-80)	84 (64-104)	83 (65-98)	78 (44-95)	64 (58-102)	62 (49-114)	0.9376	KW
FEV1 % pred.	81 (52-109)	57 (29-84)	91 (58-104)	82 (50-113)	82 (80-111)	103 (80-110)	0.8677	KW
FEV1/VC % pred.	135 (134-136)	89 (84-93)	108 (97-115)	129 (104-131)	115 (112-124)	136 (108-153)	0.0348	KW
ITGV % pred.	85 (84-86)	108 (96-119)	91 (65-132)	88 (46-105)	87 (66-92)	108 (48-127)	0.7328	KW
RV % pred.	95 (89-100)	127 (124-129)	88 (58-113)	72 (49-129)	49 (49-120)	80 (10-134)	0.6192	KW
RV/TLC % pred.	133 (108-157)	140 (122-158)	104 (69-122)	113 (87-138)	79 (70-110)	113 (18-122)	0.3513	KW
R tot kPa x s/l	0.33 (0.31-0.34)	0.19 (0.11-0.26)	0.33 (0.25-0.48)	0.41 (0.27-0.69)	0.37 (0.23-0.42)	0.34 (0.28-0.45)	0.5887	KW
DLCO % pred.	37 (17-57)	65 (49-81)	61 (33-73), n: 3	43 (28-68)	74 (61-89)	53.5 (41-66), n: 2	0.3518	KW
KCO % pred.	65, n: 1	79.5 (66-93)	66 (35-94), n: 3	65 (46-86)	92.5 (67-118), n: 2	81 (67-95), n: 2	0.4987	KW
SaO_2 in %	93.5 (91-96)	96.5 (96-97)	95.5 (92.5-96.75)	96 (94.5-97.5)	97 (96-98)	96 (95-97)	0.4846	KW
pO_2 (mmHg)	68.5 (56-81)	88.5 (87-90)	75 (65.25-83.5)	82 (72.5-90.5)	84 (79-89)	86 (74-91)	0.3282	KW
pCO_2 (mmHg)	36.5 (36-37)	41 (39-43)	39 (38.25-41.75)	37 (34.75-38.5)	41 (40-45)	44 (37-44)	0.0549	KW
LTOT, n (%)	1 (50)	0 (0)	1 (13)	1 (20)	0 (0)	0 (0)	0.5751	Chi^2

6 MWD (meters)	215 (120-310)	600, n: 1	480, n: 1	360 (270-450), n: 4		420 (360-480), n: 2	0.1853	KW
VO_2max (ml/kg/min)		18, n: 1		14 (12-25), n: 3	22 (19-28)		0.3679	KW
Finger Clubbing, n (%)	1 (50)	1 (50)	1 (12,5)	0 (0)	1 (33.3)	1 (33.3)	0.5291	Chi^2
Velcro rales, n (%)	1 (50)	0 (0)	0 (0)	1 (20)	1 (33.3)	1 (33.3)	0.4574	Chi^2
NSAID, n (%)	0 (0)	1 (50)	3 (37.5)	1 (20)	1 (33.3)	3 (100)	0.2304	Chi^2
PPI, n (%)	1 (50)	1 (50)	2 (25)	2 (40)	2 (66.6)	2 (66.6)	0.7738	Chi^2
Systemic steroids	1 (50)	1 (50)	7 (87.5)	3 (60)	2 (66.6)	1 (33.3)	0.6133	Chi^2

Abbreviations: Comparison of clinical and functional parameters for the ILD patient collective. The data are given in median (interquartile range), numbers or percentage (n, %). Calculation of p values was performed with use of Kruskal-Wallis Test (KWU), Dunnett's multiple comparison tests and One-way Anova; Chi^2-Test (Chi^2) for nominal variables, and Mann-Whitney-U (MWU) Test for BAL. If data was not available from all patients, the number (s) is specified.

Supplementary Table S2. Demographics, clinical and lung function parameters in patient cohorts underlying measurements in BALF.

	**Healthy controls n = 20**	**IPF n = 21**	**ILD n =31**	**Pooled analysis, p-value**	**Test**
Age	30 (25-58)	60 (55-65) **	55 (43-67)	0.0027	KW
Gender (♀/♂)	11/9	6/15	17/14	0.1263	Chi^2
Active smoker, n (%)	1 (5)	1 (1)	1 (8)		
Never smoked, n (%)	1 (5)	2 (9.5)	12 (39)	0.7711	Chi^2
Ex-smoker, n (%)	3 (15)	12 (57)	10 (32)		
LDH (U/I)	157 (125-195), n: 11	245 (203-313), n: 20 ***	238 (186-298), n: 29 ***	0.0001	KW
CK (U/l)	78 (65-96), n: 8	74 (49-98)	77 (51-118), n:26	0.9146	KW
CRP (mg/dl)	2.5 (0.8-4), n: 8	2 (1.3-6.6)	4 (1.6-6)	0.3312	KW
BAL: (ml)	98 (87-104), n: 8	100 (80-110)	98 (80-113)	0.9322	KW
Macrophages %	89 (83-94), n: 8	84 (69-90)	65 (41-82) **	0.0031	KW
Neutrophils %	3.5 (1-5), n: 8	5 (2.5-9.5)	4 (2-19)	0.5202	KW
Eosinophils %	1 (0-1), n: 8	3 (1-4) *	1 (0-2.25), n: 30	0.0155	KW
Lymphocytes %	5.5 (4.25-12), n: 8	6 (2.5-14.5)	19 (6-36) *	0.0133	KW
CD4/CD8 ratio	4.3, n: 1	2.3 (1.4-4.6), n: 11	4.8 (0.9-9), n: 23	0.4706	KW
VC % pred.	95 (91-116), n: 8	64 (54-72), n: 20**	71 (55-92) **	0.0035	KW
FVC % pred.	97 (84-119), n: 6	67 (53-75)	70 (56-94), n: 30	0.014	KW
TLC % pred.	103 (95-126), n: 8	69 (56-82), n: 20 ***	89 (71-105) ***	0.0001	KW
FEV1 % pred.	98 (89-109), n: 8	71 (55-77), n: 20 **	74 (59-87) **	0.003	KW

FEV1/VC % pred.	107 (97-112), n: 8	110 (102-118), n: 20	104 (98-110), n: 30	0.2606	KW
ITGV % pred.	104 (88-129), n: 6	67 (55-87), n: 20 **	85 (72-108)	0.0015	KW
RV % pred.	139 (110-148), n: 8	75 (66-100), n: 20 ***	111 (92-148)	< 0.0001	KW
RV/TLC % pred.	117 (97-137), n: 6	110 (98-122), n: 20 *	133 (109-146), n: 30 *	0.0293	KW
R tot kPa x s/l	88 (80-103), n: 8	86 (69-100) **	110 (93-133)	0.01	KW
DLCO % pred.	69 (63-75), n: 2	43 (34-52), n: 19 **	55 (44-83), n: 27	0.01	KW
KCO % pred.	77, n:1	65 (59-81), n: 20	82 (61-97), n: 26	0.3105	KW
SaO_2 in %	95 (95-95), n: 3	94 (93-96), n: 20	94 (92-95), n: 30	0.6495	KW
pO_2 (mmHg)	77 (72-92), n: 5	70 (66-74), n: 19	67 (60-75), n: 26	0.0489	KW
pCO_2 (mmHg)	37 (34-41), n: 5	38 (36-41), n: 19	38 (35-41), n: 26	0.7014	KW
LTOT, n (%)	0 (0)	4 (20)	6 (19) ***	0.0003	Chi^2
6 MWD (meters)		405 (352-477)	406 (314-454), n: 26	0.6377	MWU
VO_2max (ml/kg/min)		13.7 (12.2-18), n: 15	16.4 (13.4-20.4), n: 21	0.2414	MWU
NYHA Grade I-IV		3 (2-3), n: 15	2 (2-3), n: 15	0.1551	MWU
BORG Score in 6 MWD		3 (2-6), n: 19*	1 (1-3), n: 25	0.04	MWU
Digital Clubbing, n (%)	0 (0)	10 (14)***	4 (31)	0.0003	Chi^2
Velcro rales, n (%)	0 (0)	17 (24) ***	17 (55)***	< 0.0001	Chi^2
NSAID, n (%)	0 (0)	4 (19)	8 (26)	0.051	Chi^2
PPI, n (%)	2 (10)	3 (14)	11 (35)	0.0594	Chi^2
Systemic steroids, n(%)	0 (0)	7 (33)	8 (26) *	0.0211	Chi^2

Abbreviations: The data are given in median (interquartile range), numbers or percentage (n, %). The NSIP, HP and sarcoidosis are grouped together as ILDs. If data was not available from all patients, the number (s) is specified. Calculation of p values was performed with use of Kruskal-Wallis Test (KW), Dunnett's multiple comparison tests and One-way Anova; Chi^2-Test (Chi^2) for nominal variables, and Mann-Whitney-U Test (MWU). * - $p < 0.05$, ** - $p < 0.01$, *** - $p < 0.0001$.
